# Thioredoxin-1 Protects against Neutrophilic Inflammation and Emphysema Progression in a Mouse Model of Chronic Obstructive Pulmonary Disease Exacerbation

**DOI:** 10.1371/journal.pone.0079016

**Published:** 2013-11-11

**Authors:** Naoya Tanabe, Yuma Hoshino, Satoshi Marumo, Hirofumi Kiyokawa, Susumu Sato, Daisuke Kinose, Kazuko Uno, Shigeo Muro, Toyohiro Hirai, Junji Yodoi, Michiaki Mishima

**Affiliations:** 1 Departments of Respiratory Medicine, Graduate School of Medicine, Kyoto University, Kyoto, Japan; 2 Louis Pasteur Center for Medical Research, Kyoto, Japan; 3 Department of Biological Responses, Institute for Virus Research, Kyoto University, Kyoto, Japan; 4 Center for Cell Signaling Research and Department of Bioinspired Science, Ewha Womans University, Seoul, Korea; University of Rochester Medical Center, United States of America

## Abstract

**Background:**

Exacerbations of chronic obstructive pulmonary disease (COPD) are characterized by acute enhancement of airway neutrophilic inflammation under oxidative stress and can be involved in emphysema progression. However, pharmacotherapy against the neutrophilic inflammation and emphysema progression associated with exacerbation has not been established. Thioredoxin-1 has anti-oxidative and anti-inflammatory properties and it can ameliorate neutrophilic inflammation through anti-chemotactic effects and prevent cigarette smoke (CS)-induced emphysema. We aimed to determine whether thioredoxin-1 can suppress neutrophilic inflammation and emphysema progression in a mouse model of COPD exacerbation and if so, to reveal the underlying mechanisms.

**Results:**

Mice were exposed to CS and then challenged with polyinosine-polycytidylic acid [poly(I:C)], an agonist for virus-induced innate immunity. Airway neutrophilic inflammation, oxidative stress and lung apoptosis were enhanced in smoke-sensitive C57Bl/6, but not in smoke-resistant NZW mice. Exposure to CS and poly(I:C) challenge accelerated emphysema progression in C57Bl/6 mice. Thioredoxin-1 suppressed neutrophilic inflammation and emphysema progression. Poly(I:C) caused early neutrophilic inflammation through keratinocyte-derived chemokine and granulocyte-macrophage colony-stimulating factor (GM-CSF) release in the lung exposed to CS. Late neutrophilic inflammation was caused by persistent GM-CSF release, which thioredoxin-1 ameliorated. Thioredoxin-1 enhanced pulmonary mRNA expression of MAP kinase phosphatase 1 (MKP-1), and the suppressive effects of thioredoxin-1 on prolonged GM-CSF release and late neutrophilic inflammation disappeared by inhibiting MKP-1.

**Conclusion:**

Using a mouse model of COPD exacerbation, we demonstrated that thioredoxin-1 ameliorated neutrophilic inflammation by suppressing GM-CSF release, which prevented emphysema progression. Our findings deepen understanding of the mechanisms underlying the regulation of neutrophilic inflammation by thioredoxin-1 and indicate that thioredoxin-1 could have potential as a drug to counteract COPD exacerbation.

## Introduction

Chronic obstructive pulmonary disease (COPD) is an inflammatory condition involving oxidative stress and various types of inflammatory cells such as neutrophils and macrophages [1], [2]. It is the fourth leading cause of death worldwide [1]. The degree of pulmonary emphysema, which is a major pathological change in COPD, correlates with lung function [3], [4] and prognosis [5]. Cigarette smoke (CS) is the most important risk factor for emphysema [1], but emphysema can progress even after some patients with COPD stop smoking [6]. A pharmacological intervention to sufficiently regulate inflammation in COPD and to prevent emphysema progression has not yet been established. Indeed, chronic inflammation under conditions of oxidative stress is relatively resistant to corticosteroids that comprise the standard anti-inflammatory treatment [7], [8].

Exacerbation of COPD, which is clinically defined as a sudden worsening of COPD symptoms, is characterized by acute enhancement of airway inflammation [9], [10], [11], oxidative stress [11] and proteolysis [12], and further amplification of neutrophilic inflammation is a prominent feature [9]. Exacerbation negatively affects mortality [13] and lung function [14]. Moreover, we previously showed that emphysema progression involves exacerbations [15], the prevention and treatment of which are quite important for COPD management. Clinical trials have shown that systemic corticosteroid therapy can improve clinical status and lung function in the short term [16], [17], [18], However, our previous findings have suggested that current standard treatment regimens including systemic corticosteroids might not sufficiently suppress exacerbation-induced, long-term emphysema progression [15]. It remains unclear whether acute-on-chronic inflammation during exacerbation can be sufficiently regulated by systemic corticosteroid, although chronic inflammation in stable state of COPD has been previously shown to poorly respond to corticosteroids [8]. Thus, not only the effects and limitations of corticosteroids, but also the potential of alternative therapeutics in exacerbation of COPD should be investigated.

Thioredoxin-1 (TRX) is a ubiquitous, redox-acting, small protein of 105 amino acids with a conserved CXXC construct in its active site that exchanges dithiol to disulfide to maintain the redox status of other molecules [19], [20], [21]. In addition to this anti-oxidative effect, TRX has anti-inflammatory [22], [23], [24] and anti-apoptotic properties [25]. TRX overexpression and recombinant TRX administration are effective in animal models of many diseases such as emphysema and acute respiratory distress syndrome [26], [27], [28]. TRX inhibits neutrophil chemotaxis induced by lipopolysaccharide [22] and CS [26] and thus it could be a candidate drug for treating COPD exacerbation characterized by airway neutrophilic inflammation and emphysema progression [9], [15].

Viral infection is a major cause of COPD exacerbation [29], [30]. Studies have shown that viral infection in mice exposed to CS enhances lung inflammation similar to that in humans [31], [32], [33], and this enhancement can be mimicked by administration of polyinosine-polycytidylic acid (poly(I:C)), a synthetic double stranded RNA that is an agonist for innate immunity to viral infection [32], [33]. Moreover, these poly(I:C) challenges accelerate emphysema progression in CS-exposed mice. This model can be used for investigating the immune-pathological changes seen in human COPD exacerbations.

We postulated that recombinant TRX suppresses the excessive inflammatory response, especially neutrophilic inflammation, and subsequent emphysema progression induced by COPD exacerbation. We therefore evaluated the effects of TRX in the mouse model of COPD exacerbation and the underlying mechanisms involved.

## Materials and Methods

The Animal Research Committee of Kyoto University approved the study protocols.

### Animals and Exposure to Cigarette Smoke

Male C57Bl/6NCrSlc and NZW mice purchased from Japan SLC (Shizuoka, Japan) were housed in a temperature-controlled conventional room and freely supplied with laboratory chow and water for at least 3 weeks before being exposed to CS. Eleven-week-old mice were exposed to CS of 10 filter-cut standard cigarettes (Kentucky 3R4F reference cigarette, Cigarette Laboratory at the Tobacco and Health Research Institute, University of Kentucky, Lexington, KY, USA) for 50 minutes per day for 5 days per week for 22, 24, and 45 days using a nose-breathing exposure system (SG- 200; Shibata Scientific Technology Ltd., Tokyo, Japan) [26]. CS was prepared with a standard puff of 35 ml volume and 2 puffs per minute, and diluted to 3% with compressed air. Blood carboxy-hemoglobin levels were about 10% immediately after exposure and the concentration of total particulate matter in mainstream CS was 512.6 mg/m^3^.

### Poly(I:C) Challenge

**Figure 1:**
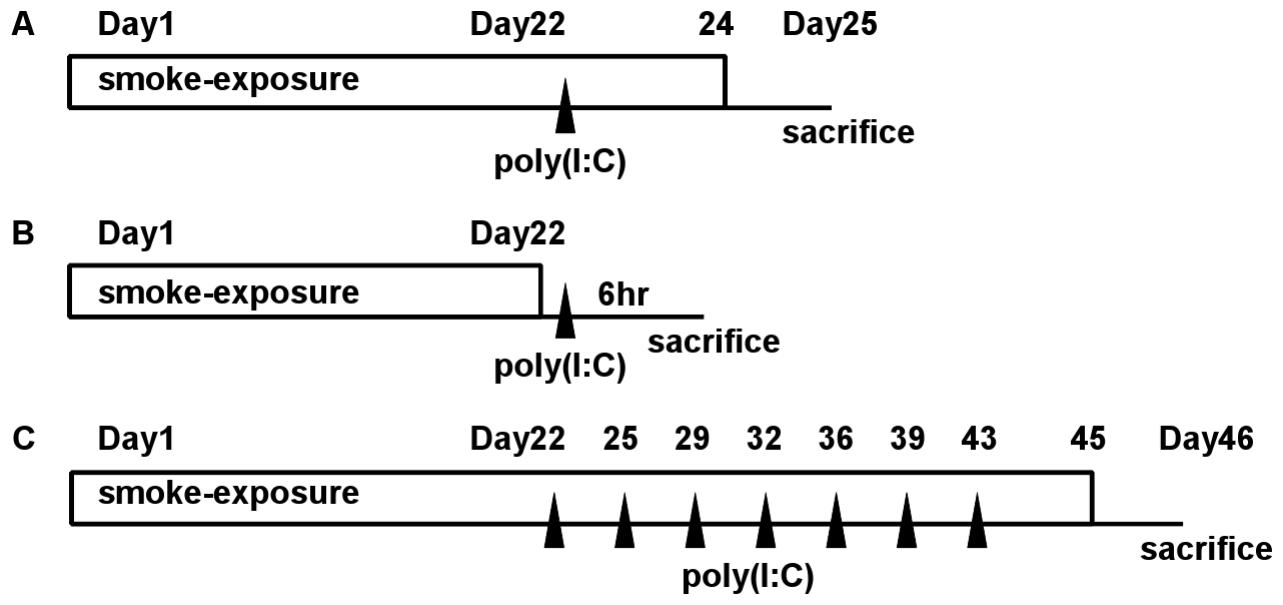
Time course of cigarette smoke exposure and poly(I:C) challenge. (A) Mice were exposed to cigarette smoke (CS) or air to day 24. Poly(I:C) or saline was challenged on day 22, and mice were sacrificed on day 25. (B) In CS-exposed mice, poly(I:C) was challenged on day 22. CS exposure was continued to day 22, and mice were sacrificed 6 hours after the challenge. (C) CS- or air-exposed C57Bl/6 mice were challenged with poly(I:C) or saline seven times (days 22, 25, 29, 32, 36, 39, and 43). CS or air exposure was continued to day 45, and mice were sacrificed on day 46.

Under light anesthesia with isoflurane, 1 mg/kg (body weight) of poly(I:C) (Sigma Aldrich, St. Louis, MO, USA) in 100 microliter of saline was administered by oropharyngeal aspiration [34]. Figure 1 summarized duration of exposure to CS and time course of poly(I:C) challenges for each experimental protocol. In single challenge experiments, poly(I:C) was administered 4 h after exposure to CS on day 22. Some groups of mice were exposed to CS to day 24 and sacrificed on day 25 (3 days after the poly(I:C) challenge, Figure 1A), while the remaining were sacrificed 6 hours after the challenge (Figure 1B). In repeated challenge experiments for lung morphometry, poly(I:C) was also administered 4 h after exposure to CS on days 22, 25, 29, 32, 36, 39, and 43. CS exposure was continued to day 45, and mice were sacrificed on day 46 (Figure 1C).

### Treatment with Systemic Corticosteroid, TRX, Anti- granulocyte-macrophage Colony-stimulating Factor (GM-CSF) Antibody, and NSC

Dexamethasone (DEX; D2915, Sigma Aldrich) was intraperitoneally injected 1 h before poly(I:C) challenge at doses of 0.1, 0.3 and 1.0 mg/kg. At 1 h before and 3 h after poly(I:C) challenge, 4 mg/kg of recombinant human TRX (Redox Bioscience Inc., Kyoto, Japan) was intraperitoneally injected. To determine the effect of GM-CSF on airway neutrophil inflammation induced by CS combined with poly(I:C), rat anti-mouse GM-CSF antibody (R&D Systems, Abingdon, UK) was delivered to the lung by oropharyngeal aspiration 3 h after poly(I:C) challenge. To investigate the effects of inhibition of dual-specificity phosphatase 1 [MAP kinase phosphatase 1 (MKP-1)], 2 mg/kg of the cell-permeable, quinone-based, dual-specificity phosphatase inhibitor, NSC 95397 (#N1786, Sigma Aldrich) was intraperitoneally injected both 1.5 h before and 4 h after the poly(I:C) challenge.

### Bronchoalveolar Lavage (BAL)

Three days after a single administration of poly(I:C), mice were anesthetized with 20 mg/kg of intraperitoneal pentobarbital. Lungs were lavaged through an intratracheal cannula twice with 1 mL of cold saline and then the inflammatory cell differential, inflammatory cytokines and oxidative stress in the airway were assessed in BAL fluid (BALF). Inflammatory cytokines were also measured 6 h after poly(I:C) challenge in another experiment. The BALF was centrifuged and inflammatory cell differential was determined (Shandon Scientific Ltd., Runcorn, Cheshire, UK). Supernatants were stored at −80°C. At least 400 cells were counted on each cytospin slide stained with Diff-Quik (Dade Behring, Inc., Deerfield, IL, USA) under a light microscope.

### Protein Carbonyls and Inflammatory Cytokines in BALF

Protein carbonyl (a marker of oxidative stress) and inflammatory cytokines were measured using Protein Carbonyl Enzyme Immuno-Assay kits (BioCell Corporation Ltd., Papatoetoe, New Zealand) and Bioplex (Bio-Rad Laboratories, Richmond, CA, USA), respectively. Levels of GM-CSF were measured using Bioplex assay and ELISA kit (R&D Systems, Abingdon, UK).

### Tissue Preparation

Right lungs were frozen in liquid nitrogen and stored for mRNA and protein analysis. Left lungs were inflated with 50% optimal cutting temperature fluid at 25 cm of H_2_O pressure and frozen in cold isopentane for immunohistochemistry and morphometry. Frozen sections (7 µm thick) were cut using a Cryostat (Thermo Fisher Scientific, Tokyo, Japan).

### RNA Isolation and Real-time PCR

Total RNA was isolated from lung homogenates using Trizol (Invitrogen, Carlsbad, CA, USA). Gene transcripts of MMP-9, MMP-12, GM-CSF (CSF2), MKP-1 (DUSP-1), MKP-3 (DUSP-3), and 18S as an endogenous control were quantified using the ABI 7300 Real-Time PCR System (Applied Biosystems, Foster City, CA, USA) with oligonucleotide PCR primer pairs and fluorogenic probes (TaqMan Gene Expression Assay; Applied Biosystems).

### Microarrays

Total RNA samples were pooled for each experimental group and analyzed using the 3D-Gene™ Mouse Oligo chip 24 k (Toray Industries Inc., Tokyo, Japan) and then gene expression ratios of TRX-treated to non-treated mice were calculated. The expression array data are deposited in Gene Expression Omnibus under accession number GSE49450.

### Immunohistochemistry

Frozen lung sections were incubated with anti-single-stranded DNA (ssDNA) antibody (1∶2000 dilution; Dako North America Inc., Carpinteria, CA, USA) and anti-cleaved caspase-3 antibody (1∶200 dilution; Cell Signaling, Danvers, MA, USA) [26], [35]. Sections were stained using the Dako EnVision system (peroxidase/DAB; Dako, Kyoto, Japan). Immunoreactive cells are expressed as ratios of positive cell to the length of the alveolar septa.

### Morphometry

Frozen lung sections were stained with Diff-Quik and assessed by investigators who were blinded to the status of the animals. The extent of emphysema was evaluated as mean linear intercept (Lm), destructive index (DI), and as the standard deviation (SD) and coefficient of variation (CV) of terminal airspace sizes as described [26], [36]. Lm and DI were manually measured in at least 10 fields. The original microscope images were converted into binary images and each contiguous air space was automatically identified using custom software to calculate the SD and CV of terminal airspace sizes [36] (Figures S1 and S2).

### Statistics

Results are expressed as means ± SD. Data were statistically analyzed using JMP 7 software (SAS Institute, Cary, NC). Groups were compared by analysis of variance followed by the Tukey-Kramer or Dunnett’s *post hoc* test. *P<*0.05 was considered significant.

## Results

### Comparison of Poly(I:C) Impact in Mice with Different Susceptibilities to CS-induced Emphysema After Exposure to CS

**Figure 2:**
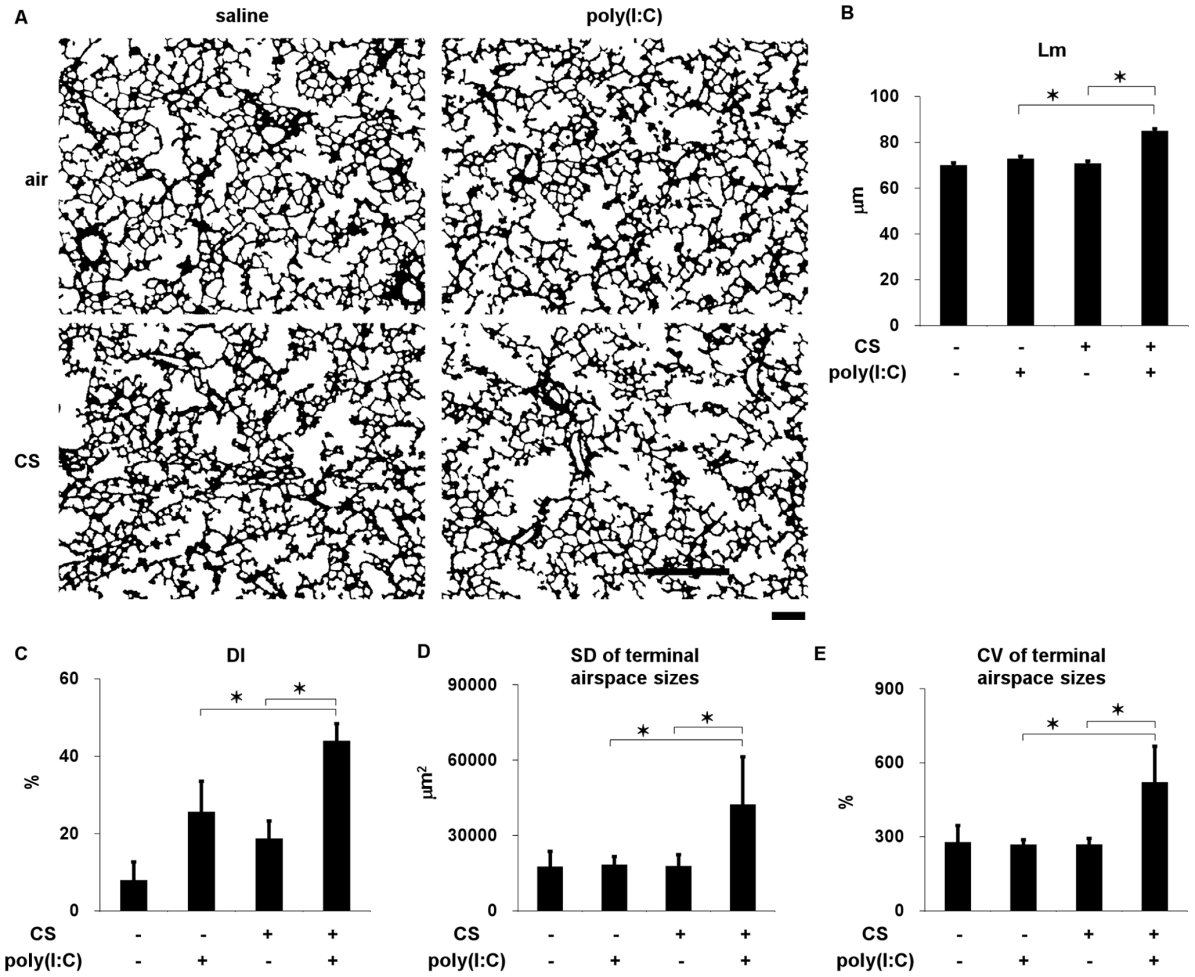
Lung morphometry in C57Bl/6 mice cigarette exposed to cigarette smoke or air and challenged with poly(I:C) or saline seven times. (A) Representative binary images of lung photomicrographs (×4). (B) Mean linear intercept (Lm). (C) Destructive index (DI). (D) Standard deviation (SD) and (E) coefficient of variation (CV) of terminal airspace sizes. Scale bar, 200 µm. Error bars represent SD (n = 5–6 per group); *p<0.05.

To determine the effects of CS and poly(I:C) on the progression of emphysema, C57Bl/6 mice, which are susceptible to the development of CS-induced emphysema [37], were exposed to CS or air for forty-five days. Poly(I:C) or saline was administered into the lungs seven times (days 22, 25, 29, 32, 36, 39, and 43) (Figure 1C). The Lm, DI, SD and CV in the terminal airspace sizes were significantly increased in the mice exposed to CS and poly(I:C) (Figure 2 and Figure S1), indicating that this combination of agents contributed to airspace enlargement, the destruction of alveolar walls and increased spatial heterogeneity, which is a structural feature of progressive emphysema [38].

**Figure 3:**
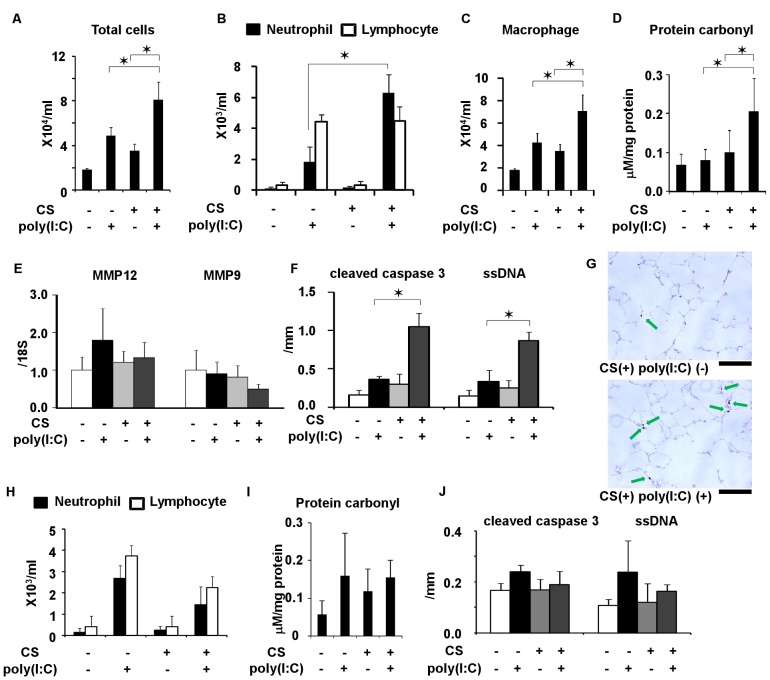
Comparison of impact of poly(I:C) between C57Bl/6 and NZW mice exposed to cigarette smoke. (A) Total cell counts, (B) neutrophil and lymphocyte counts, and (C) macrophage count, and (D) protein carbonyl in BALF. (E) mRNA expression of MMP-12 and MMP-9 in lung homogenates and (F) cleaved caspase 3- and ssDNA-positive cells in lung sections from C57Bl/6 mice exposed to cigarette smoke or air and challenged with poly(I:C) or saline once. (G) Representative images showing cleaved caspase 3-positive cells (arrow) in the lungs of C57BL/6 mice (×20). Scale bar, 100 µm. (H) Neutrophil and lymphocyte counts in BALF, (I) protein carbonyl in BALF, and (J) cleaved caspase 3- and ssDNA-positive cells in lung sections from NZW mice. Error bars represent standard deviation (SD) (n = 5–6 per group); *p<0.05.

To identify components enhanced by CS and poly(I:C), C57Bl/6 mice were exposed to CS or air for three weeks and then administered with poly(I:C) or saline once (Figure 1A). The counts of total cells neutrophils, and macrophages, but not of lymphocytes, were significantly increased in BALF by CS and poly(I:C) (Figure 3A B, and C). The levels of protein carbonyl in BALF and the numbers of cleaved caspase 3- and ssDNA-positive cells (markers of apoptosis) in the lungs were also significantly increased (Figure 3D, F, and G), whereas MMP-9 and MMP-12 mRNA induction was not affected (Figure 3E). Poly(I:C) combined with CS did not increase protein carbonyl levels or total cell, neutrophil, and macrophage counts in BALF, or apoptotic cell markers in the lungs of NZW mice that are resistant to developing emphysema induced by CS [37] (Figure 3F, G and H). Therefore, we considered that these components were exacerbation-related, rather than general non-specific changes caused by viral infections and that they could feasibly be used to evaluate responses to therapy in this model.

### Effects of TRX and Systemic Corticosteroids on Poly(I:C)-induced Changes in C57Bl/6 Mice Exposed to CS

**Figure 4:**
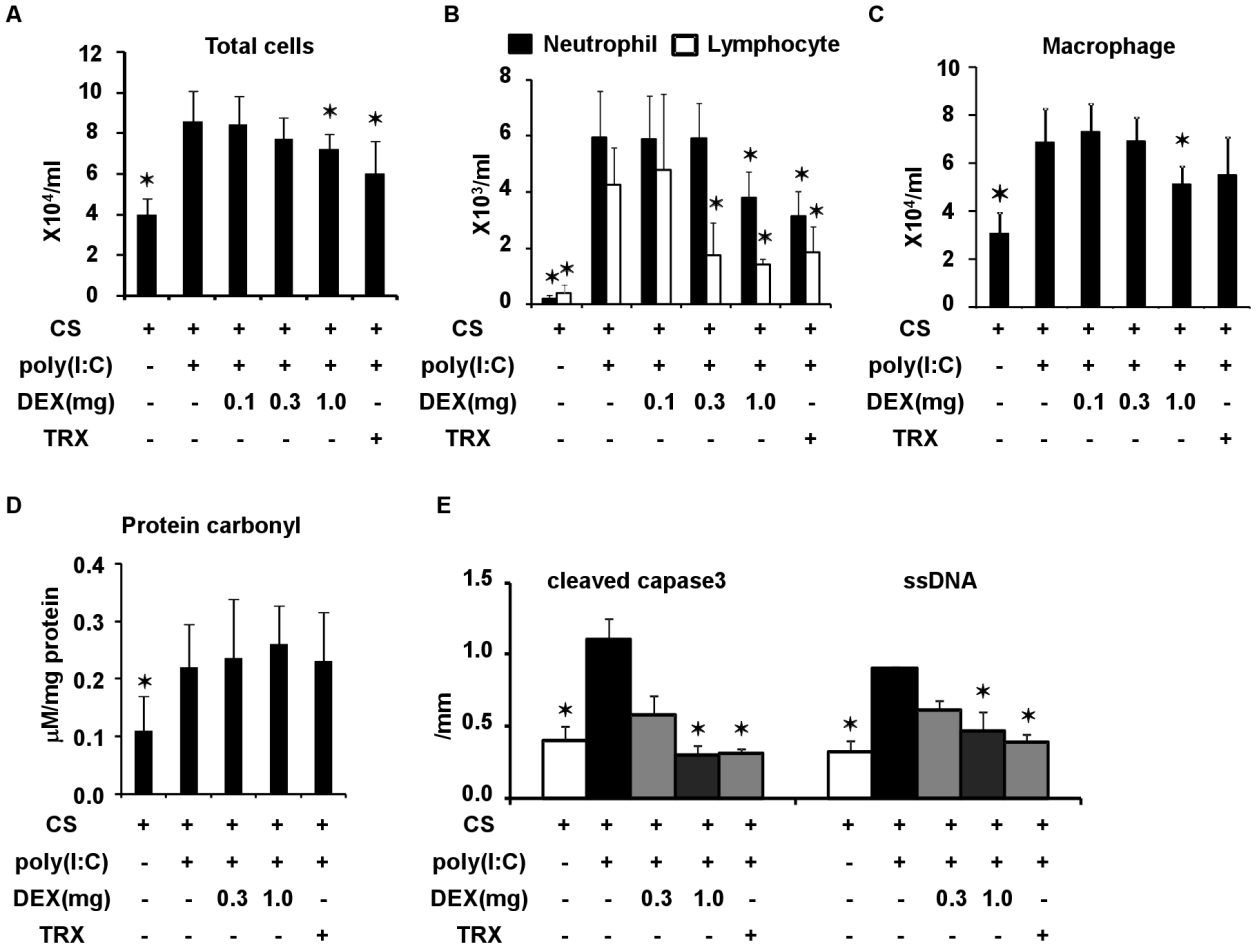
Effects of dexamethasone (DEX) at 0.1, 0.3, and 1 mg/kg and TRX in C57Bl/6 mice exposed to cigarette smoke and challenged with poly(I:C). (A) total cell counts, (B) neutrophil and lymphocyte counts, (C) macrophage count, and (D) protein carbonyl in BALF, and (E) cleaved caspase 3- and ssDNA-positive cells in lung sections. Error bars represent standard deviation (SD) (n = 4–6 per group; *p<0.05 compared with untreated mice exposed to cigarette smoke and poly(I:C).

After three weeks of exposure to CS, poly(I:C) was administered together with an intraperitoneal injection of TRX and various doses of DEX or saline. Duration of CS exposure and time course of poly(I:C) challenge was shown in Figure 1A and B. At 1 h before and 3 h after the poly(I:C) challenge, 4 mg/kg of TRX was intraperitoneally injected. DEX (0.1, 0.3 and 1.0 mg/kg) was intraperitoneally injected 1 h before the poly(I:C) challenge. Total counts of cells and neutrophils in BALF 3 days after the poly(I:C) challenge were significantly decreased by TRX, as well as by 1.0, but not ≤0.3 mg/kg of DEX (Figure 4A and B). Macrophages in BALF were significantly decreased by 1.0 mg/kg of DEX, but not by TRX (Figure 4C). Levels of protein carbonyl in BALF were not decreased by TRX or DEX at any dose (Figure 4D). Cleaved caspase-3-positive cells and ssDNA-positive cells were significantly reduced by TRX and 1.0 mg/kg of DEX (Figure 4E).

### Effects of TRX and Systemic Corticosteroids on Lung Morphometry in C57Bl/6 mice Exposed to CS and Poly(I:C)

**Figure 5:**
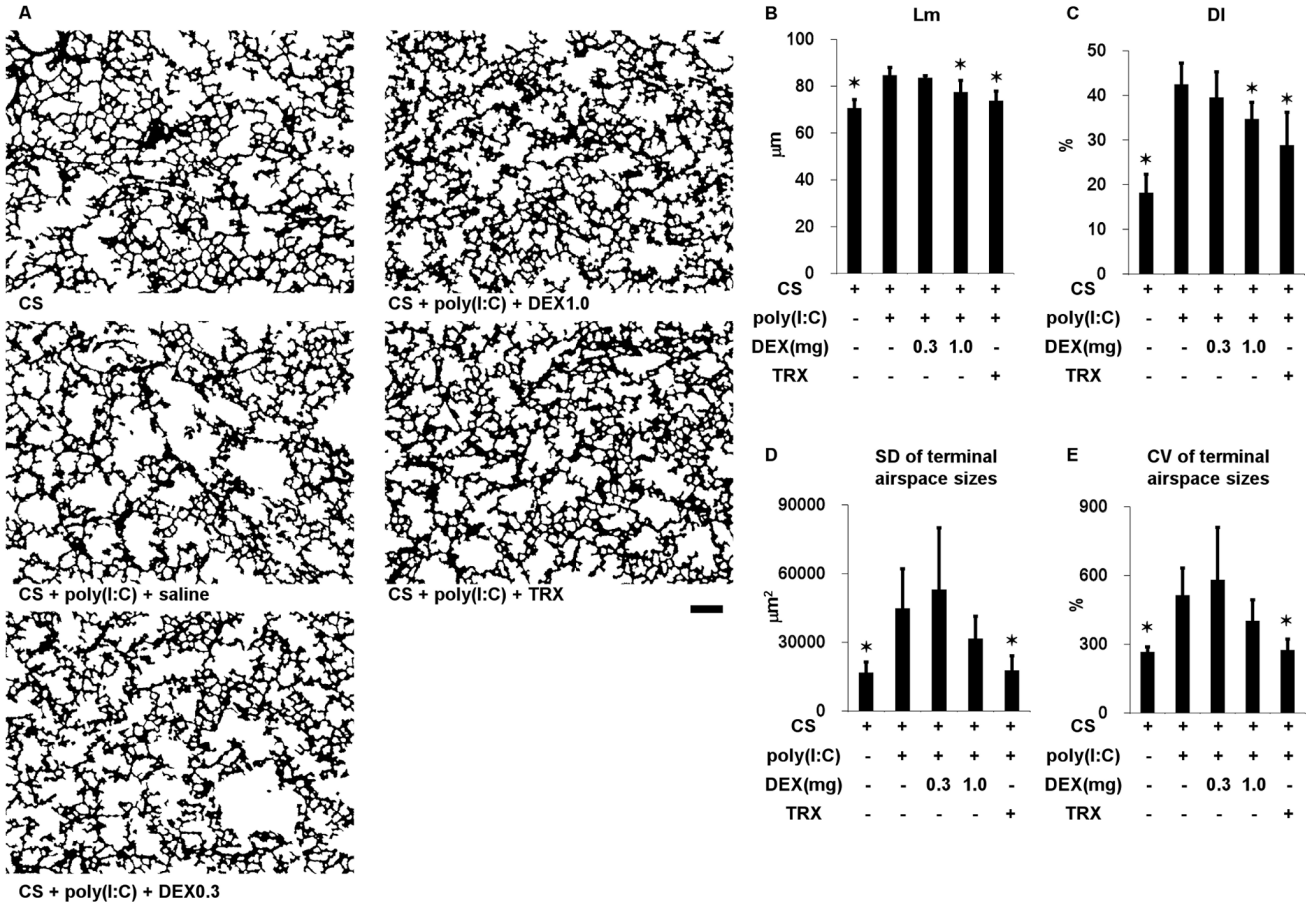
Lung morphometry in mice exposed to cigarette smoke and in those exposed to cigarette smoke, challenged with poly(I:C) and treated with dexamethasone (DEX) at 0.3 and 1 mg/kg, TRX or saline. (A) Representative binary images of lung photomicrographs (×4). (B) Mean linear intercept (Lm). (C) Destructive index (DI). (D) Standard deviation (SD) and (E) coefficient of variation (CV) of terminal airspace sizes. Scale bar, 200 µm. Error bars represent SD (n = 5–6 per group); *p<0.05 compared with mice exposed to cigarette smoke, challenged with poly(I:C) and treated with saline.

We administered poly(I:C) seven times along with TRX, DEX (0.3 or 1.0 mg/kg) or saline in mice exposed to CS to determine lung morphometry. Duration of CS exposure and time course of poly(I:C) challenges was shown in Figure 1C. Challenge with poly(I:C) significantly increased the Lm, DI, and SD and CV of terminal airspace sizes (Figure 5 and Figure S2). The increases in Lm and DI were significantly prevented by TRX and by 1.0, but not by 0.3 mg/kg of DEX. TRX significantly ameliorated the increases in the SD and the CV of terminal airspace sizes, whereas DEX at all tested doses did not.

### Anti-inflammatory Effect of TRX

**Figure 6:**
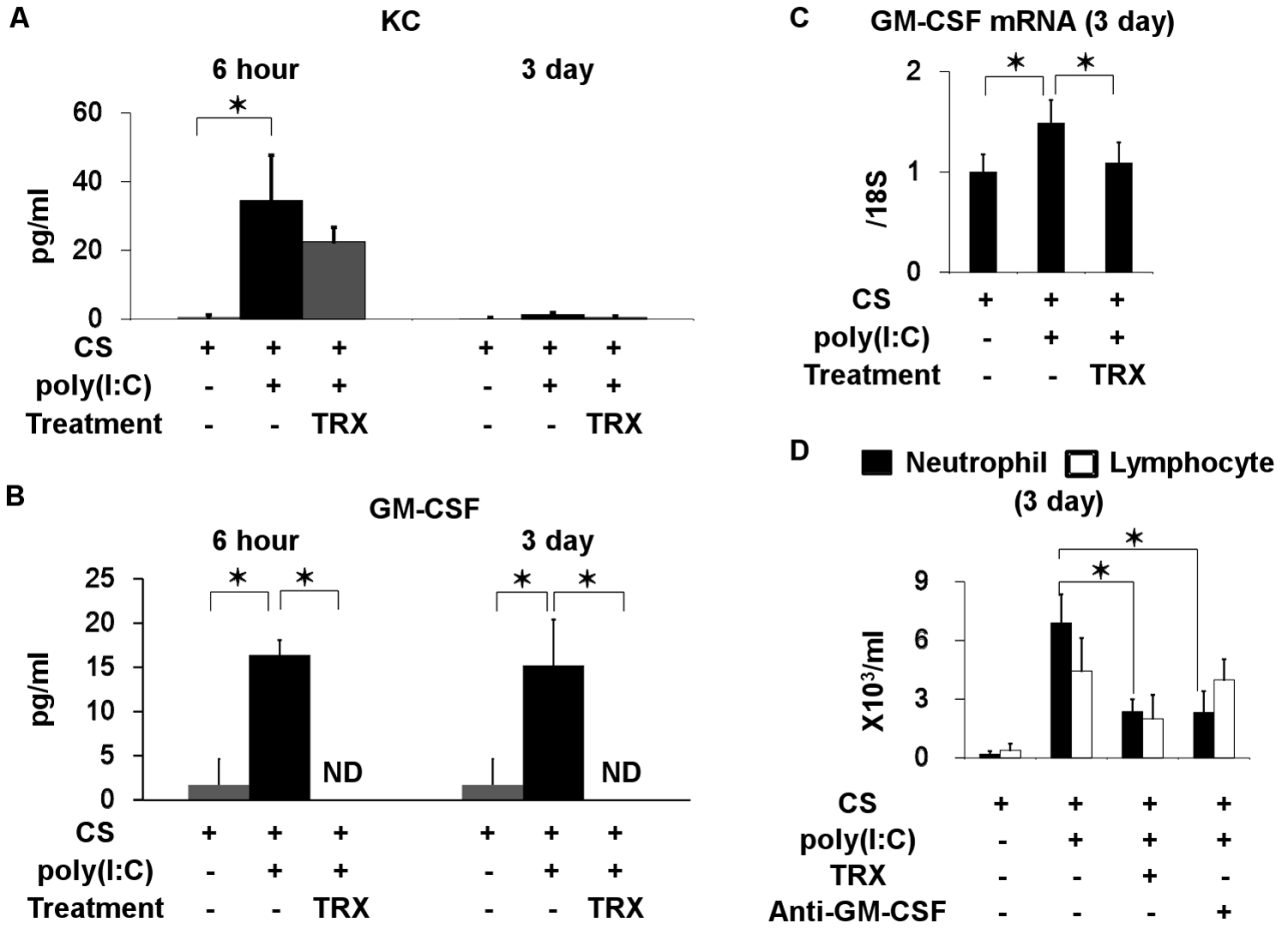
Effects of TRX on inflammatory cytokines in BALF from exposed to cigarette smoke and challenged with poly(I:C). (A) KC and (B) GM-CSF in BALF obtained 6 h and 3 days after poly(I:C) challenge. (C) Messenger RNA of GM-CSF in lung homogenates 3 days after poly(I:C) challenge in mice exposed to cigarette smoke treated with or without TRX. (D) Neutrophil and lymphocyte counts in BALF 3 days after poly(I:C) challenge in mice exposed to cigarette smoke treated with and without TRX or anti-GM-CSF antibody. Error bars represent standard deviation (SD) (A, B, and C, n = 3–4 per group; D and E, n = 5 per group); *p<0.05.

We investigated how TRX regulates airway neutrophilic inflammation by measuring levels of inflammatory cytokines in BALF. Many cytokines, including neutrophil chemokines such as keratinocyte-derived chemokine (KC) and GM-CSF, were significantly increased at 6 h after poly(I:C) challenge (Table S1). Notably, the increase in GM-CSF was still detectable after 3 days, whereas that in KC spontaneously resolved (Figure 6A and B and Table S2). TRX ameliorated the sustained increase in GM-CSF 3 days after the challenge. Moreover, the significantly increased mRNA level of GM-CSF at 3 days after poly(I:C) challenge in lung homogenates of mice exposed to CS was ameliorated by TRX (Figure 6C). The neutrophil count in BALF in mice exposed to CS at 3 days after poly(I:C) challenge was significantly and similarly decreased by aspirated anti-GM-CSF antibody and TRX (Figure 6D).

### Transcriptional Regulation by TRX in Mice Exposed to CS and Poly(I:C)

**Figure 7:**
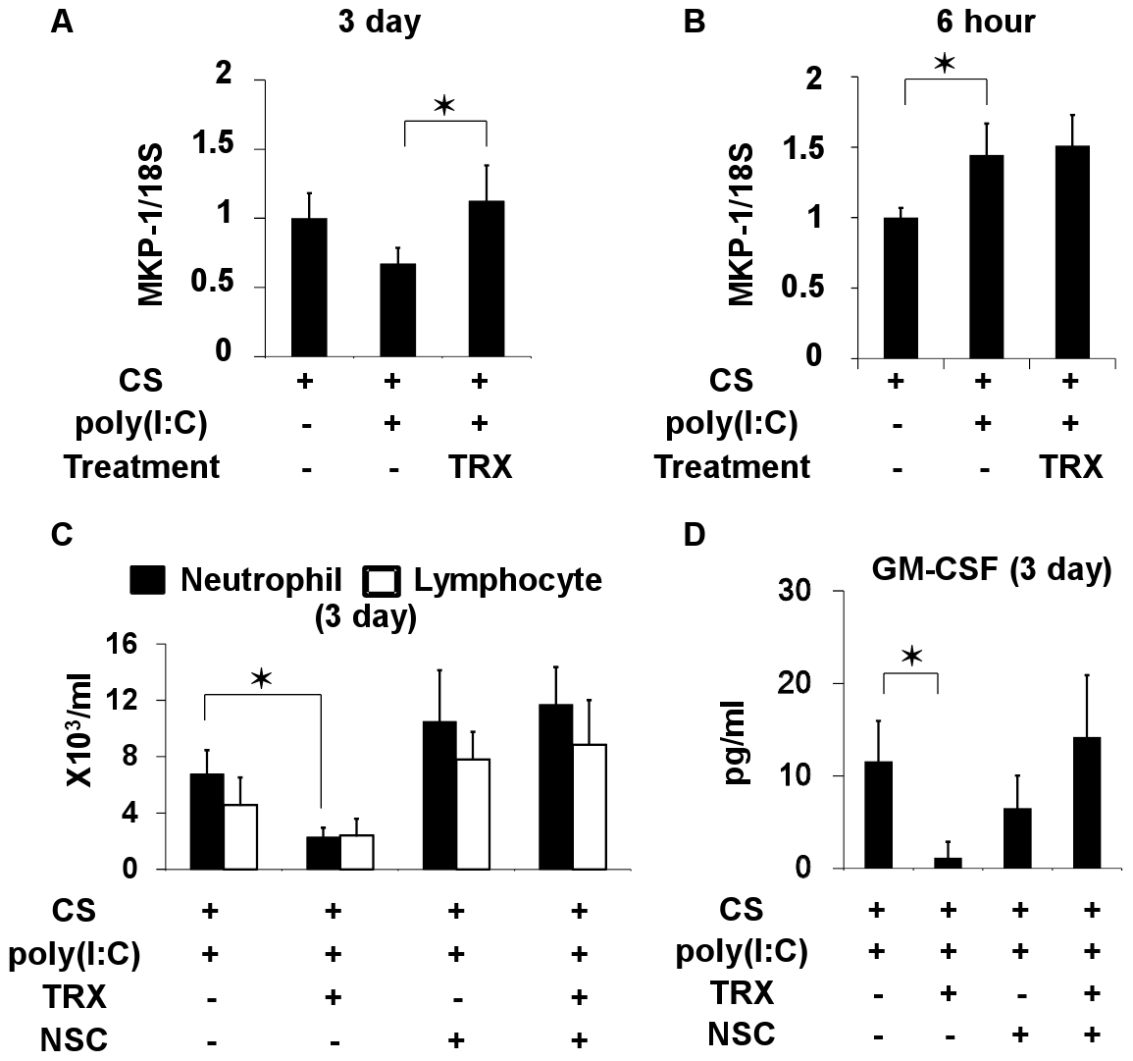
Pulmonary mRNA expression of MKP-1 in mice exposed to cigarette smoke then challenged with poly(I:C), and profiles of inflammatory cells and GM-CSF in BALF from mice treated with MKP-1 inhibitor. (A) MKP-1 mRNA in mice treated with TRX and saline at 3 days after poly(I:C) challenge. (B) MKP-1 mRNA at 6 h after poly(I:C) challenge. (C) Profiles of inflammatory cells and (D) GM-CSF levels in BALF at 3 days after poly(I:C) challenge from mice exposed to cigarette smoke and treated with or without TRX or cell-permeable, quinone-based, dual-specificity phosphatase inhibitor, NSC 95397. Error bars represent standard deviation (SD) (A and B, n = 3–4 per group; D and E, n = 5 per group); *p<0.05.

To identify a candidate molecule involved in anti-inflammatory effects of TRX, the expression profiles of pulmonary mRNA in mice exposed to CS and poly(I:C) and then treated or not with TRX were examined using microarrays. Among possible genes that were up- or down- regulated by TRX (data not shown), dual-specificity phosphatase 1, also called MAP kinase phosphatase 1 (MKP-1) was further investigated because TRX inhibits P38 MAP kinase in neutrophils [22] and MKP-1 negatively regulates inflammatory responses both *in vitro* and *in vivo* [39], [40]. The results of real-time PCR showed that MKP-1 mRNA levels significantly increased in the lungs of mice exposed to CS at 3 days (Figure 7A), but not at 6 h (Figure 7B), after the poly(I:C) challenge and treatment with TRX compared with saline.

We investigated whether MKP-1 up-regulation is associated with the suppressive effect of TRX on airway neutrophil inflammation and GM-CSF production by inhibiting MKP-1 using NSC 95397, which inhibits both MKP-1 and MKP-3 [41], [42]. Unlike MKP-1, the extent of MKP-3 induction at both 6 h and 3 days after the poly(I:C) challenge in mice exposed to CS did not differ between TRX and saline treatment (Figure S3). TRX reduced BALF neutrophil counts and GM-CSF levels at 3 days after the poly(I:C) challenge in mice exposed to CS, but not in those treated with NSC95397 (Figure 7C and D).

## Discussion

The present study showed that TRX has potential to counteract neutrophilic inflammation and emphysema progression in a mouse model of COPD exacerbation. Recombinant TRX suppressed the accelerated progression of emphysema in smoke-sensitive mice exposed to CS and repeatedly challenged with poly(I:C).

**Figure 8:**
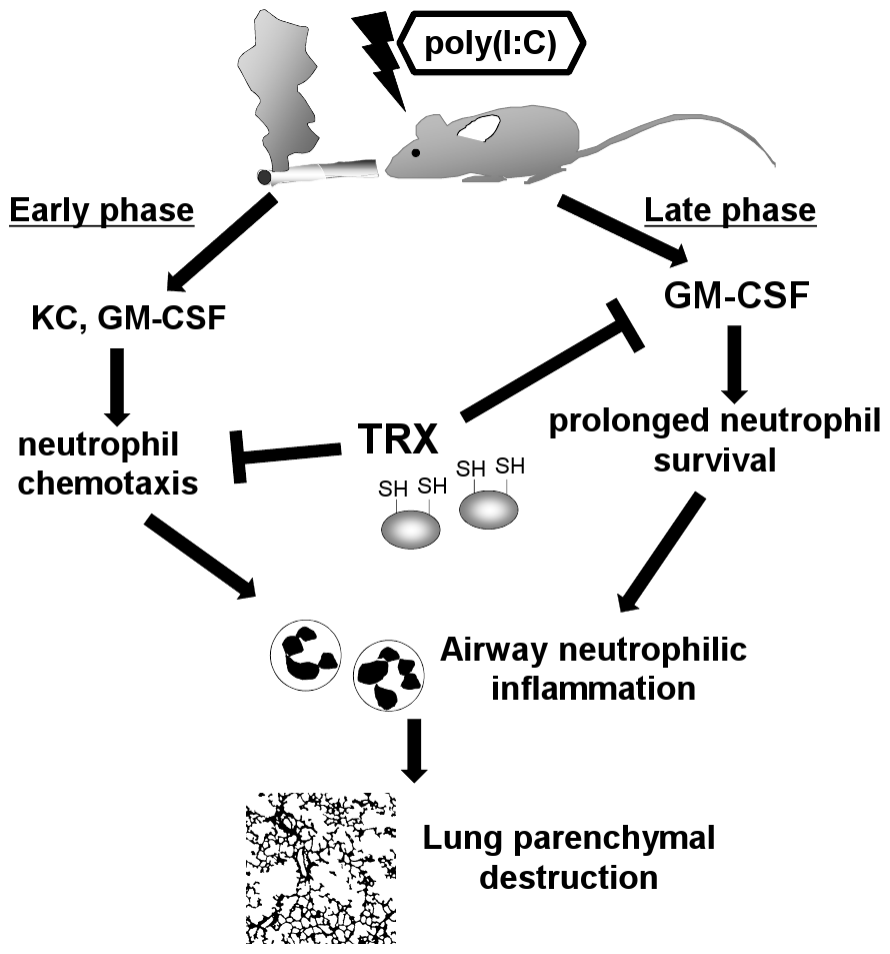
Estimated mechanism of dual regulation of poly(I:C)-induced neutrophilic inflammation by recombinant TRX in mouse lungs exposed to cigarette smoke. Poly(I:C)-induced neutrophilic inflammation consists of two phases. Poly(I:C) induces neutrophilic chemokines such as KC and GM-CSF that cause neutrophil migration into the lung during the early phase. Thereafter, sustained release of GM-CSF in the lung contributes to prolong neutrophil survival, resulting in persistent airway inflammation throughout the late phase. Thioredoxin-1 suppresses airway neutrophil inflammation through directly inhibiting neutrophil chemotaxis and reducing GM-CSF.

Our findings deepen understanding of the mechanism underlying the regulation of neutrophilic inflammation by TRX. Exaggerated airway neutrophilic inflammation was central to the accelerated progression of CS and poly(I:C)-induced emphysema, and neutrophilic inflammation comprised two phases (Figure 8). Poly(I:C)-induced production of neutrophilic chemokines such as KC and GM-CSF promoted neutrophil migration into the lung during the early phase, and then the sustained release of GM-CSF in the lung prolonged neutrophil survival [43] during the late phase, which led to persistent airway inflammation and pronounced parenchymal destruction. TRX can suppress neutrophilic inflammation, perhaps through directly inhibiting neutrophil infiltration into sites of inflammation [22]. Notably, we discovered that TRX suppresses prolonged GM-CSF release, indicating that recombinant TRX regulates neutrophilic inflammation via a dual mechanism.

We used the animal model established by Kang et al. [32] with slight modification. It has been shown that in this model, airway inflammation is greater and emphysema develops more rapidly than conventional mouse model of emphysema induced by CS exposure alone. This model is appropriate for exploring enhanced airway inflammation and accelerated emphysema progression, which are the main immunopathological changes in human COPD exacerbation [9], [10], [15]. Although the time course of poly(I:C) challenge and duration of smoke exposure slightly differed in the present, from the original study, similar inflammatory responses and progressive emphysema were detected (lung cell apoptosis and parenchymal destruction).

To identify “exacerbation-related changes”, we compared smoke-sensitive C57Bl/6 mice (murine counterpart of patients with COPD) and smoke-resistant NZW mice (murine counterpart of asymptomatic smokers) assuming that changes induced by poly(I:C) in NZW mice were not related to exacerbation. Consequently, exposure to CS and poly(I:C) enhanced airway neutrophilic and macrophage inflammation and induced oxidative stress and lung apoptosis in smoke-sensitive, but not in smoke-resistant, mice. We considered that these findings were exacerbation-related changes that should be targeted with therapeutic interventions. It should be also noted that although various types of inflammatory cells such as neutrophil, macrophage, and T cells are associated with the pathogenesis of murine emphysema induced by CS alone [44], neutrophil and macrophage play an important role in amplifying airway inflammation in the present COPD exacerbation model.

TRX suppressed airway neutrophilic inflammation, lung apoptosis and the further progression of emphysema in mice exposed to CS and poly(I:C). Although TRX has anti-oxidant properties, these were not considered central in the present model because TRX did not improve the increase in oxidative stress assessed by carbonyl protein in BALF. This finding was consistent with previous reports concerning the limited anti-oxidant properties of exogenous TRX [45]. To reinforce this conclusion, other markers of oxidative stress such as F2-isoprostanes should be measured [46].

GM-CSF is a direct neutrophil chemotactic factor that increases neutrophil survival in the respiratory tract, and can be involved in CS-induced airway neutrophilic inflammation [43], [47]. The present study showed that in mice exposed to CS and poly(I:C), the airway level of GM-CSF was increased at 6 h after poly(I:C) challenge and sustained until 3 days after the challenge, while the levels of other inflammatory cytokines especially associated with neutrophilic inflammation including KC, IL-6, RANTES, and TNF alpha were increased at 6 h after poly(I:C) challenge but spontaneously resolved at 3 days after the challenge. These results indicate that early phase of CS and poly(I:C)-induced neutrophilic inflammation could be prompted by many cytokines, but the enhanced inflammation could be sustained exclusively by prolonged GM-CSF release.

TRX ameliorated enhanced GM-CSF mRNA expression and protein production at 3 days after poly(I:C) challenge. The airway neutrophil inflammation at 3 days after the challenge was reduced as much by anti-GM-CSF antibody as by TRX in mice exposed to CS. These suggest that TRX regulates late phase of neutrophilic inflammation by suppressing prolonged GM-CSF release. The suppressive effects of TRX against the early increases in inflammatory cytokines such as IL-6, TNF alpha, and RANTES were also found, and this might have affected the reduction of GM-CSF and resolution of neutrophilic inflammation during the late phase.

To elucidate the signaling pathway associated with the suppression of GM-CSF release and regulation of neutrophilic inflammation by treatment with TRX, we focused on MKP-1 in the lung of mice treated with TRX based on the findings of an investigation into the suppressive effect of TRX on P38 MAP kinase in neutrophils [22]. Inflammatory cytokine release is regulated by MKP-1 in innate immune responses [39], [40].

Pulmonary mRNA of MKP-1 was up-regulated at 6 h after poly(I:C) challenge in both mice exposed to CS and then treated with TRX or saline, but the extent of MKP-1 induction did not differ between the two groups. In contrast, 3 days after the challenge, more MKP-1 was expressed in the group treated with TRX than with saline. TRX reduced neutrophil counts and GM-CSF levels in BALF at 3 days after poly(I:C) challenge in mice exposed to CS, but this effect disappeared in mice exposed to CS and treated with the MKP-1 and MKP-3 inhibitor NSC95397 [41], [42]. These findings suggest that MKP-1 might be involved in the suppression of GM-CSF release and late phase of neutrophilic inflammation by TRX.

In addition to mRNA expression, we examined MKP-1 protein levels in the lungs at 3 days after poly(I:C) challenge using Western blotting. However, MKP-1 protein levels did not significantly differ between mice treated with or without TRX (data not shown). This is a major limitation of the present study. Nevertheless, our findings are quite important, because they show for the first time an association between TRX, MKP-1, and inflammation. The findings also provide a hypothesis that MKP-1 induction by TRX is essential for suppressing persistent GM-CSF release and neutrophilic inflammation. This should be verified in future studies.

Effects of systemic corticosteroids on “exacerbation-related changes” such as airway neutrophilic inflammation and emphysema progression were also evaluated. In human, 30–40 mg/body (approximately 0.5–0.67 mg/kg) of prednisolone has been recommended for treatment of COPD exacerbations [1]. Given that 0.75 mg/kg of DEX is equivalent in anti-inflammatory activity to 5 mg/kg of prednisolone, 0.1 mg/kg of DEX in the present model could be relevant to the clinical dose currently applied to manage COPD exacerbation. Notably, airway neutrophilic inflammation and emphysema progression could be suppressed only when the dose of DEX was increased up to 1.0 mg/kg, which may reflect approximately 10 times of the standard dose in practice. These suggest that the current regimen of systemic corticosteroids cannot always prevent emphysema progression induced by exacerbation. Together with concern that high dose of systemic corticosteroid has risk of adverse effects, our results emphasize the importance of further investigation about the role of TRX as alternative therapeutics.

We found that many inflammatory cytokines such as IL-6, TNF alpha, and RANTES in BALF were increased and pulmonary mRNA of MKP-1 were up-regulated at 6 h after poly(I:C) challenge in CS-exposed mice. Since MKP-1 negatively regulates inflammatory cytokines such as IL-6 and TNF alpha [40], [48], it is possible that the early up-regulation of MKP-1 acts as negative feedback regulator leading to the spontaneous reductions in IL-6, RANTES, and TNF alpha at 3 days after the challenge.

Some limitations are associated with this study. Poly(I:C) challenges proceeded before emphysema was established. The present model reflects exacerbations during the early, but not the moderate to severe stages of COPD. However, a distinct subgroup of patients with COPD can experience frequent exacerbations independently of disease severity [49]. We believe that the present model provides information about the immune-pathological changes that are qualitatively similar to those in COPD patients.

Our animal model of COPD exacerbation was established using poly(I:C), and not a virus infection and thus the influence of pharmacological intervention on viral clearance or the adaptive immune response in exacerbations could not be assessed.

In the present study, after identifying “exacerbation-related changes” by using CS- or air-exposed mice challenged with saline or poly(I:C), effects of TRX and DEX were evaluated only in CS-exposed mice, but not in air-exposed mice, because the main aim of the present study was to investigate effects of TRX against acute-on-chronic inflammation and lung parenchymal destruction during exacerbation, and because mice exposed to CS and then challenged with or without poly(I:C) were considered as murine counterpart of exacerbation or stable state of COPD, respectively. However, considering that inflammation under oxidative stress generally shows a poor response to corticosteroid [7], [8], it is also an important issue whether effects of TRX and DEX against poly(I:C)-induced inflammation might differ between mice exposed to CS and air. This should be investigated in future studies.

In conclusion, airway neutrophilic inflammation and the progression of emphysema was suppressed by TRX and a relatively high dose, but not by a moderate dose of systemic corticosteroid in smoke-sensitive model mice exposed to poly(I:C) and CS. Our findings also suggest a novel mechanism of neutrophilic inflammation regulated by TRX. In addition to the inhibition of neutrophil chemotaxis, the suppression of prolonged GM-CSF release by TRX is involved in the resolution of late phase of poly(I:C)-induced neutrophilic inflammation. The present findings suggest that TRX has a dual regulatory effect on neutrophilic inflammation induced by poly(I:C) in the lungs of model mice exposed to CS and indicate that TRX has potential as a novel therapeutic agent for treating COPD exacerbation.

## Supporting Information

Figure S1 **Representative original images (Diff-Quik), binary images, and color map images that identify each terminal airspace in cigarette smoke- or air-exposed C57Bl/6 mice challenged with poly(I:C) or saline seven times (magnification ×4).** Scale bar, 200 um.
(TIF)

Figure S2 **Representative original images (Diff-Quik), binary images, and color map images that identify each terminal airspace in cigarette smoke-exposed poly(I:C)-challenged mice treated with different doses of dexamethasone (DEX; 0.3 and 1 mg/kg), TRX, and saline (magnification ×4).** Scale bar, 200 um.
(TIF)

Figure S3 **Pulmonary mRNA expressions of MKP-3 in cigarette smoke-exposed mice challenged with poly(I:C) once.** (A) MKP-3 mRNA in mice treated with TRX and saline 6 hours after poly(I:C) challenge. (B) MKP-3 mRNA 3 days after the poly(I:C) challenge. Error bars represent standard deviation (SD) (n = 3–4 per group).
(TIF)

Table S1 **Cytokine levels in bronchoalveolar lavage fluid of cigarette smoke-exposed mice treated with thioredoxin or saline 6 hours after poly(I:C) challenge.**
(DOC)

Table S2 **Cytokine levels in bronchoalveolar lavage fluid of cigarette smoke-exposed mice treated with thioredoxin or saline 3 days after poly(I:C) challenge.**
(DOC)
