# Supplementary figures and images for: Thioredoxin-1 Protects against Neutrophilic Inflammation and Emphysema Progression in a Mouse Model of Chronic Obstructive Pulmonary Disease Exacerbation
Source: PLoS One. 2013 Nov 11;8(11):e79016. doi: 10.1371/journal.pone.0079016 (PMC3823967; doi:10.1371/journal.pone.0079016)

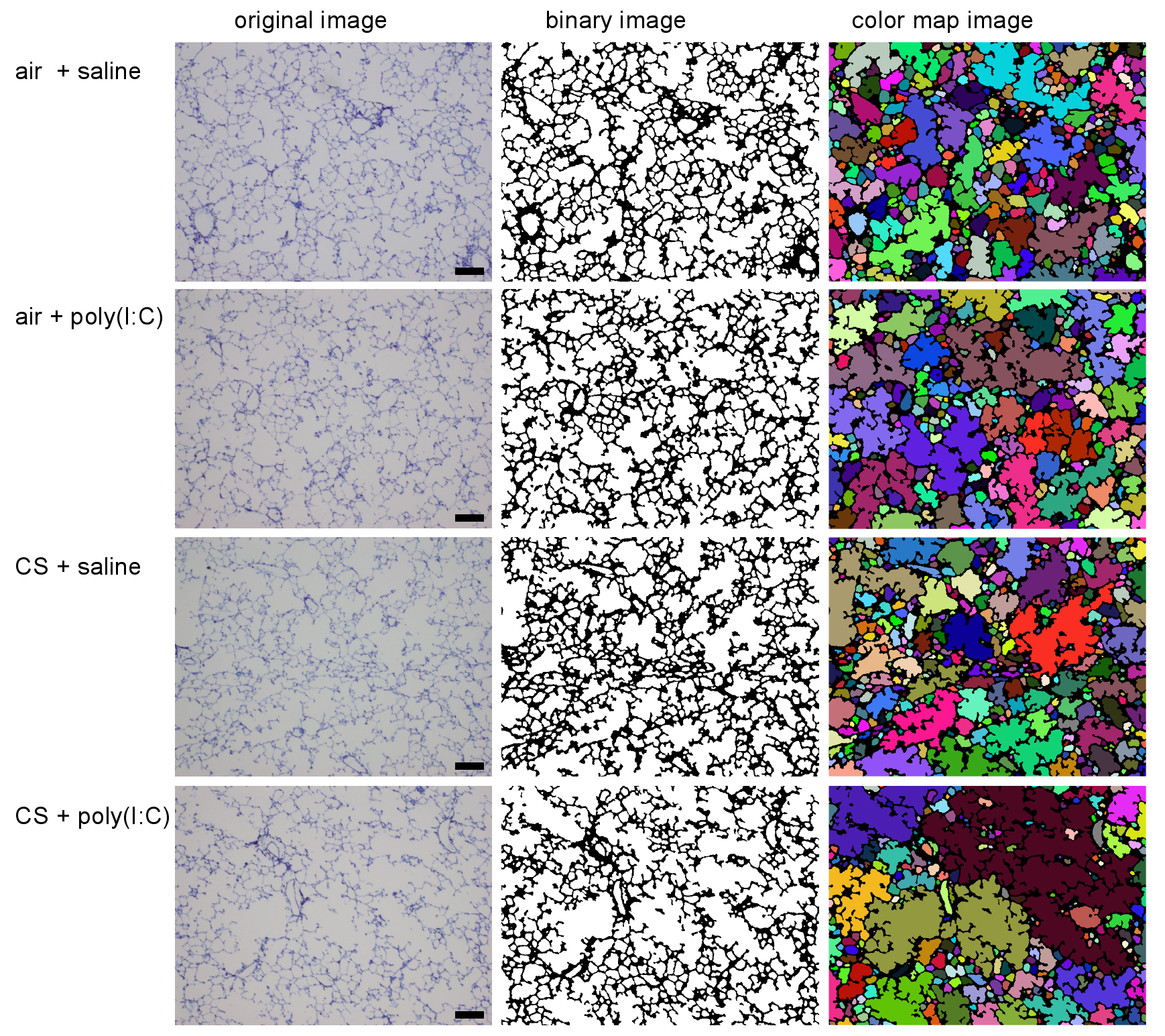

Supplement: Figure S1 — Representative original images (Diff-Quik), binary images, and color map images that identify each terminal airspace in cigarette smoke- or air-exposed C57Bl/6 mice challenged with poly(I:C) or saline seven times (magnification ×4). Scale bar, 200 um. (TIF) [file pone.0079016.s001.tif]

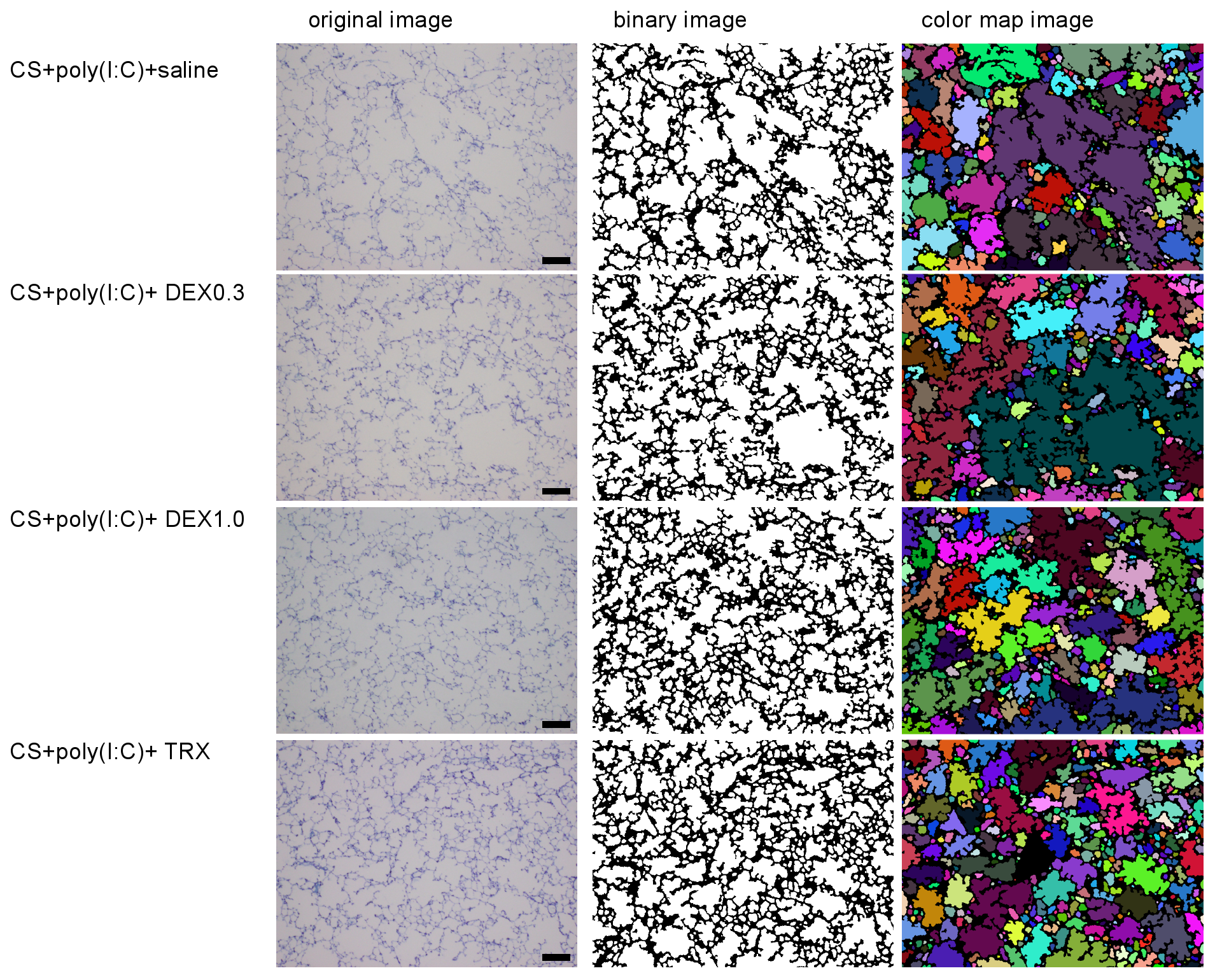

Supplement: Figure S2 — Representative original images (Diff-Quik), binary images, and color map images that identify each terminal airspace in cigarette smoke-exposed poly(I:C)-challenged mice treated with different doses of dexamethasone (DEX; 0.3 and 1 mg/kg), TRX, and saline (magnification ×4). Scale bar, 200 um. (TIF) [file pone.0079016.s002.tif]

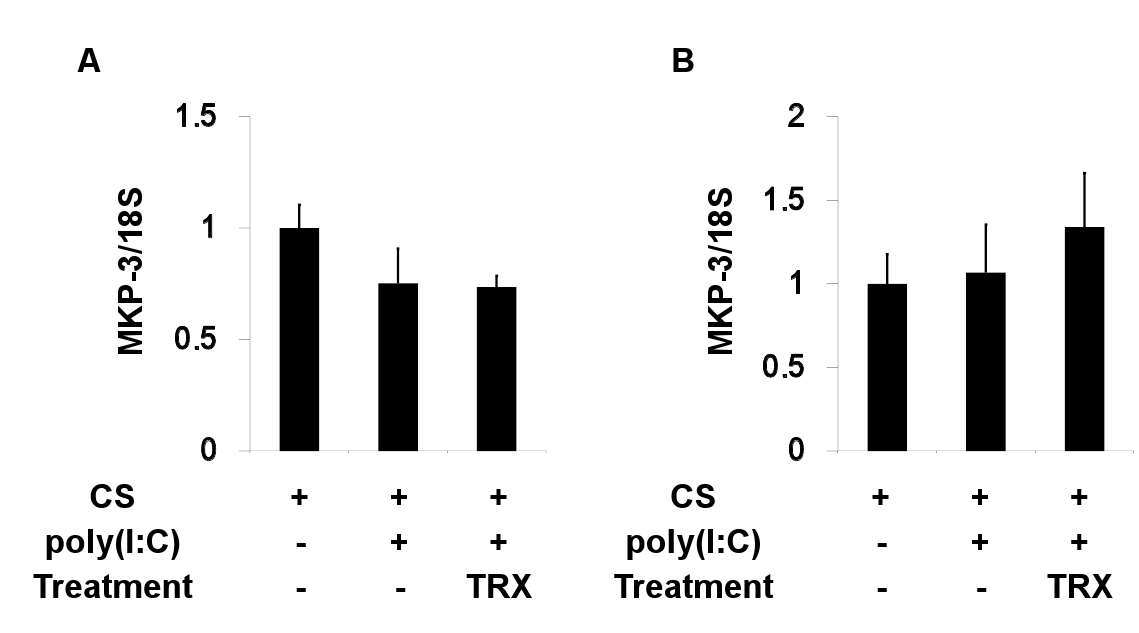

Supplement: Figure S3 — Pulmonary mRNA expressions of MKP-3 in cigarette smoke-exposed mice challenged with poly(I:C) once. (A) MKP-3 mRNA in mice treated with TRX and saline 6 hours after poly(I:C) challenge. (B) MKP-3 mRNA 3 days after the poly(I:C) challenge. Error bars represent standard deviation (SD) (n = 3–4 per group). (TIF) [file pone.0079016.s003.tif]
