# Supplementary material for: Thioredoxin-1 Protects against Neutrophilic Inflammation and Emphysema Progression in a Mouse Model of Chronic Obstructive Pulmonary Disease Exacerbation
Source: PLoS One. 2013 Nov 11;8(11):e79016. doi: 10.1371/journal.pone.0079016 (PMC3823967; doi:10.1371/journal.pone.0079016)
Supplement: Table S1 — Cytokine levels in bronchoalveolar lavage fluid of cigarette smoke-exposed mice treated with thioredoxin or saline 6 hours after poly(I:C) challenge. (DOC) [file pone.0079016.s004.doc]

**Table S1. Cytokine levels in bronchoalveolar lavage fluid of cigarette smoke-exposed mice treated with thioredoxin or saline** 6 hours after poly(I:C) challenge

|  | CS  n=3 | CS+poly(I:C)+Saline  n=4 | CS+poly(I:C)+TRX  n=4 |
| --- | --- | --- | --- |
| IL-1alpha | 0.9 (1.5) | ND | 1.4 (2.5) |
| IL-1beta | 8.7 (2.0) ***** | 37.7 (3.8) | 31.3 (3.7) |
| IL-2 | ND | ND | ND |
| IL-3 | ND | ND | ND |
| IL-4 | ND | ND | ND |
| IL-5 | ND | ND | ND |
| IL-6 | **0.1 (0.1) *** | 1081.9 (187.9) | **443.6 (75.5) *** |
| IL-9 | ND | ND | ND |
| IL-10 | 12.4 (3.8) | 11.8 (3.1) | 6.9 (1.8) |
| IL-12 (p40) | **5.1 (1.2) *** | 52.0 (5.8) | 61.9 (9.1) |
| IL-12 (p70) | 11.0 (2.2) | 1.7 (2.7) | 8.1 (7.0) |
| IL-13 | 4.2 (5.1) | 6.4 (3.5) | 3.1 (3.4) |
| IL-17 | ND | ND | ND |
| Eotaxin | ND | ND | ND |
| G-CSF | **3.3 (1.6) *** | 785.9 (114.9) | 773.7 (32.7) |
| GM-CSF | **1.7 (2.9) *** | 16.4 (1.6) | **ND*** |
| IFN-gamma | 0.0 (0.1) | 2.7 (2.5) | ND |
| KC | **0.6 (0.6) *** | 34.5 (13.2) | 22.5 (4.2) |
| MCP-1 | **18.1 (16.5) *** | 130.3 (31.6) | 94.1 (17.0) |
| MIP-1alpha | **25.0 (5.9) *** | 377.9 (74.) | 337.5 (256) |
| MIP-1beta | **ND*** | 22.9 (9.9) | 12.2 (4.9) |
| RANTES | **ND*** | 423.6 (67.1) | **258.8 (35.0) *** |
| TNFalpha | **2.7 (0.3) *** | 128 (24.) | **34.8 (21.5) *** |

Data are expressed as mean (SD). CS, cigarette smoke; ND, not detected. * p<0.05 compared to CS and poly(I:C)-exposed mice treated with saline.
