# Supplementary material for: Thioredoxin-1 Protects against Neutrophilic Inflammation and Emphysema Progression in a Mouse Model of Chronic Obstructive Pulmonary Disease Exacerbation
Source: PLoS One. 2013 Nov 11;8(11):e79016. doi: 10.1371/journal.pone.0079016 (PMC3823967; doi:10.1371/journal.pone.0079016)
Supplement: Table S2 — Cytokine levels in bronchoalveolar lavage fluid of cigarette smoke-exposed mice treated with thioredoxin or saline 3 days after poly(I:C) challenge. (DOC) [file pone.0079016.s005.doc]

**Table S2. Cytokine levels in bronchoalveolar lavage fluid of cigarette smoke-exposed mice treated with thioredoxin or saline** 3 days after poly(I:C) challenge

|  | CS  n=3 | CS+poly(I:C) +Saline  n=4 | CS+poly(I:C)+TRX  n=4 |
| --- | --- | --- | --- |
| IL-1alpha | 0.9 (1.2) | 1.4 (0.6) | 1.8 (1.6) |
| IL-1beta | 8.7 (1.5) | 16. (5.0) | 8.5 (5.9) |
| IL-2 | ND | ND | ND |
| IL-3 | ND | ND | ND |
| IL-4 | ND | ND | ND |
| IL-5 | ND | ND | ND |
| IL-6 | ND | 0.7 (0.1) | **0.2 (0.2) *** |
| IL-9 | ND | ND | ND |
| IL-10 | 12.4 (4.1) | 16.1 (1.2) | 10.7 (3.5) |
| IL-12 (p40) | 4.1 (2.0) | 9.0 (4.6) | **1.7 (0.8) *** |
| IL-12 (p70) | 12.0 (8.6) | 20.8 (0.0) | 17.2 (10.9) |
| IL-13 | 2.5 (2.4) | 5.4 (2.3) | 3.1 (3.4) |
| IL-17 | ND | ND | ND |
| Eotaxin | ND | ND | ND |
| G-CSF | 1.4 (1.6) | 2.7 (0.2) | 1.5 (0.2) |
| GM-CSF | **1.7 (2.9) *** | 15.2 (5.1) | **ND *** |
| IFN-gamma | 0.6 (1.1) | 2.0 (2.0) | ND |
| KC | **0.1 (0.2) *** | 1.0 (0.0) | **0.4 (0.3) *** |
| MCP-1 | 21.9 (5.1) | 33.8 (5.6) | 20.1 (4.6) ***** |
| MIP-1alpha | **23.7 (18.0) *** | 52.1 (6.4) | **19.9 (10.7) *** |
| MIP-1beta | ND | ND | ND |
| RANTES | ND | 3.3 (0.4) | 1.7 (1.0) |
| TNFalpha | 4.7 (4.2) | 9.7 (3.6) | 2.1 (3.2) |

Data are expressed as mean (SD). CS, cigarette smoke; ND, not detected. * p<0.05 compared to CS and poly(I:C)-exposed mice treated with saline.
